# A comparative, retrospective study of peri-articular and intra-articular injection of tranexamic acid for the management of postoperative blood loss after total knee arthroplasty

**DOI:** 10.1186/s12891-016-1293-3

**Published:** 2016-10-19

**Authors:** Zhenyang Mao, Bing Yue, You Wang, Mengning Yan, Kerong Dai

**Affiliations:** 1 Shanghai Key Laboratory of Orthopaedic Implants, Shanghai Ninth People’s Hospital, Shanghai Jiaotong University School of Medicine, Shanghai, People’s Republic of China; 2 Department of Orthopaedic Surgery, Shanghai Ninth People’s Hospital, Shanghai Jiaotong University School of Medicine, 639 Zhizaoju Road, Shanghai, 200011 People’s Republic of China

**Keywords:** Total knee arthroplasty, Tranexamic acid, Peri-articular injection, Intra-articular injection, Blood loss

## Abstract

**Background:**

Intra-articular injection of tranexamic acid (TXA) is known to be effective in controlling blood loss after total knee arthroplasty (TKA). However, this method has some disadvantages, such as TXA leakage due to soft tissue release. Peri-articular injection provides an alternative to intra-articular administration of TXA. This study aimed to evaluate the effects of peri-articular injection of TXA in reducing blood loss after TKA and compare them to those of intra-articular TXA injection.

**Methods:**

This was a retrospective analysis of 127 patients who underwent primary, unilateral TKA for knee osteoarthritis in our hospital between January 2014 and December 2014. Cases were classified into 3 comparison groups: 49 patients in the peri-articular TXA group, 36 in the intra-articular group, and 42 in the control group (TXA not administered). Demographic variables, hemoglobin (Hb) measured before and after surgery, operation time, total amount of drained volume, time of removing drains, units of blood transfused peri- and postoperatively, estimated volume of blood loss, and preoperative comorbidities were retrieved from the patients’ medical charts. Statistical analyses were performed using SPSS 19.0 software.

**Results:**

There were no significant differences of demographic variables and operation time among three groups (*P* > 0.05). Compared to the control group, both TXA groups had a significantly reduced volume of blood loss, postoperative knee joint drainage, hemoglobin concentration, time of removing drains, and need for blood transfusion (*P* < 0.05). The effects of TXA were comparable for the two methods of injection (*P* > 0.05). There were no deep venous thrombosis or thromboembolic complications in any group.

**Conclusions:**

Peri-articular injection of TXA is as effective as an intra-articular injection in reducing postoperative blood loss during TKA. Both methods had a statistically significant benefit in reducing the change in Hb concentration, volume of joint drainage, and estimated volume of blood loss when compared to the control group. Peri-articular injection of TXA can significantly reduce the blood transfusion rate compared to the control group.

## Background

Major postoperative blood loss is one of the most common problems associated with total knee arthroplasty (TKA). Postoperative blood loss can range between 1000 mL and 2000 mL [1, 2], with 10–38 % of patients requiring transfusion of 1–2 units of blood [1–4]. Blood transfusions implicitly lead to the risk for infection, graft-versus-host disease, hemolysis, and transfusion-related acute lung injury [5]. In addition, blood products are expensive and in short supply. Therefore, controlling blood loss associated with TKA is an important clinical issue.

Several methods have been introduced to reduce TKA-associated blood loss. The administration of tranexamic acid (TXA), an antifibrinolytic agent, is currently a “hot topic” of research. Several studies have reported that intravenous (IV) administration of TXA significantly reduces postoperative blood loss and the need for transfusion [4, 6]. However, several medical conditions, most notably a history of deep venous thrombosis (DVT), cerebrovascular and cardiac disease, and renal failure, are commonly identified as comorbidities in patients undergoing TKA; they affect the use of IV-TXA. In addition, only a small percentage of the TXA solution administered by IV reaches the target location and tissue [7]. To address these clinical limitations, TXA has increasingly been administered via intra-articular injection (IAI) after TKA [7–12].

The safety and efficacy of IAI of TXA has been demonstrated in several studies [7–12]. However, the reported effectiveness of this method has been questioned. In particular, the volume of intra-articular TXA solution, usually reported to be 5–25 mL in previous studies [7, 13, 14], may be insufficient to immerse the anterior tissues of the knee joint when the patient is placed in a supine position during surgery. In addition, extensive soft tissue release, which is required in some cases to balance forces on the knee, may cause the injected TXA solution to leak from the joint.

A peri-articular injection (PAI), in which the TXA solution is injected into the soft tissue around the joint cavity before closure, is an alternative to an IAI of TXA. PAI also has the distinct advantage of allowing the surgeon to target areas that are vulnerable to postoperative bleeding, such as sites of soft tissue release and incisal edges in the synovial membrane. Therefore, our aim was to conduct a retrospective analysis of patients who underwent TKA at our hospital and were administered either IAI-TXA or PAI-TXA to evaluate the effectiveness of these two modes of TXA administration in controlling postoperative blood loss compared to patients who did not receive TXA.

## Methods

TXA has been used in TKA surgeries in our hospital since May 2014. Between May 2014 and September 2014, we used an IAI of TXA in TKA as previously reported [4, 15]. However, we observed leakage of the TXA solution in some cases, especially in those who needed severe soft tissue release, and we switched to PAI of TXA in the followed TKA cases. Between January 2014 and December 2014, we retrospectively reviewed medical charts to identify patients between the ages of 50 and 80 years and in whom TKA was performed as a treatment for degenerative arthritis. Preoperative comorbidities that were thought to influence either bleeding tendency or thromboembolic tendency were recorded, as well as use of anticoagulant drugs such as warfarin and clopidogrel. All anticoagulant drugs were stopped prior to surgery, and coagulation system data were repeated and found to be normal on admission. After the retrospective review of TKA cases, 127 subjects were identified (23 male and 104 female). Of these 127 cases, 49 (8 male and 41 female) received PAI-TXA, forming the PAI-TXA group; 36 (5 male and 31 female) received IAI-TXA, forming the IAI-TXA group; and 42 patients (10 male and 32 female) were not administered TXA, forming the control group.

All surgeries were performed under general anesthesia by the same surgeon, who was a specialist in TKA procedures. A pneumatic tourniquet was prepared as a precautionary measure in case of emergent situations, such as injury to an artery, but not inflated during the surgery in those cases. A midline skin incision was made, followed by a medial para-patellar capsular approach, to expose the surgical site of the knee joint. Intra-operative hemostasis was applied in standard fashion. After bone resection of the distal femur and proximal tibia, the soft tissue balancing was performed, and the appropriate type and size of knee prosthesis components were cemented, polyethylene liners were inserted, and the patella was resurfaced. An intra-articular drain was placed in situ, followed by wound closure. For peri-articular administration of TXA, the TXA solution was injected into the soft tissues around the joint cavity, 5 to 10 mL at each point, such as posterior joint capsulae synovial membrane and ligaments, especially the sites of soft tissue release and incisal edges in the synovial membrane, before the proximal and distal components of the prosthesis were implanted. For the intra-articular administration of TXA, the TXA solution was injected into the knee joint cavity after wound closure, with the drain clamped for 15 min to allow for absorption of the TXA, and then released.

The doses of TXA were comparable to concentrations reported in previous studies of IV-TXA in TKA, showing positive effects with doses of 15 to 20 mg/kg [4, 15] and concentrations of 10–100 mg/mL for topical TXA solutions [7, 9, 14, 16]. Therefore, doses of 2 g of TXA in 80 mL normal saline were used for PAI and IAI. The contraindications of use of TXA were patients with an allergy to TXA, an actual infection, any type of cancer, atrial fibrillation and angina, stroke, rheumatoid arthritis, and revision TKA.

Postoperatively, drains were removed when the 24-h volume of drainage was less than 50 mL. Hemoglobin (Hb) was measured preoperatively and at 12 h, 24 h, and 48 h postoperatively. The change in Hb was the difference between the lowest level preoperatively and postoperatively. All patients were administered a standard course of daily oral anticoagulant (Rivaroxaban; Xarelto, Bayer Schering Pharma AG, Germany) for two weeks, starting on the first postoperative day. Patients were examined daily for clinical symptoms of DVT during the hospital stay and for 6 weeks postoperatively. Doppler ultrasonography examination was applied when there was a clinical suspicion of DVT. Once Doppler confirmed DVT, therapeutic low molecular weight heparin was administered. In addition, all patients received rehabilitation exercises that included a passive knee flexibility exercise and quadriceps femoris contraction exercise before drain removal, and the patients were allowed to walk with a walker after removal of the drain. The patients were usually discharged 14 days after surgery, unless there were wound complications. Patients had regular follow up every month for the first six months and yearly thereafter.

Demographic and clinical variables were retrieved from the patients’ medical charts and included: age, sex, height, weight, body mass index, Hb measured before and after surgery to quantify change in Hb concentration preoperative platelet blood test, preoperative coagulation system data (international normalized ratio, prothrombin time, activated partial thromboplastin time), operation time, total amount of drained volume, time of removing drains, units of blood transfused peri- and postoperatively, and preoperative comorbidities. The volume of blood loss was estimated using a previously reported method [4]. The criterion for blood transfusion was an Hb level < 8 g/dL or a postoperative Hb level between 8 and 10 g/dL with clinical signs of hemodynamic instability; this criterion was consistent with the Guidelines of the American Society of Anesthesiologists [17]. We evaluated the range of motion (ROM) to determine patients’ functional outcomes at follow-up. The ROM was measured with a universal goniometer that is commonly used in clinical practice [18, 19].

Statistical analyses were performed using SPSS 19.0 software. Continuous variables were expressed as a mean ± standard deviation. Between-group differences for demographic and clinical variables were evaluated using one-way analysis of variance, and a least squared difference post-hoc test was used for variables with homogeneity of variance or Dunnett’s test was used for variables with heterogeneity of variance. Between-group differences in the distribution of men and women in each group and the number of transfusions were analyzed with cross-tabulation. A value of *P* < 0.05 was considered to be statistically significant.

## Results

**Fig. 1:**
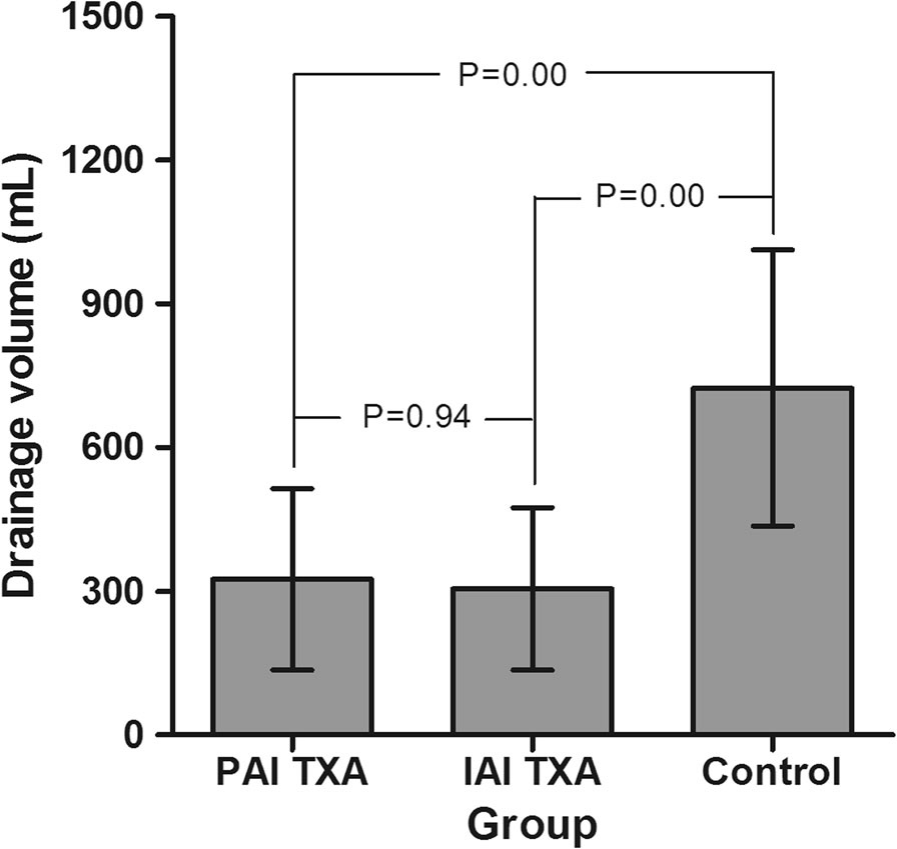
The postoperative drainage volume of the three groups. Legend: The volume was significantly lower for patients in the PAI-TXA and IAI-TXA groups compared to the control group (*P* < 0.05) and was comparable for the two TXA groups (*P* = 0.94)

The demographic variables for our study group are listed in Table 1, with no significant between-group differences among the three experimental groups. The postoperative volume of knee joint drainage is reported in Fig. 1. The volume was significantly lower for patients in the PAI-TXA and IAI-TXA groups compared to the control group (*P* < 0.05), with a mean volume of 324.9 ± 189.4 mL for the PAI-TXA group, 305.0 ± 169.1 mL for the IAI-TXA group, and 724.1 ± 288.4 mL for the control group. The volume of drainage was comparable for the two TXA groups (*P* = 0.94). The times of removing drains were 36.6 ± 12.0 h for the PAI-TXA group, 42.6 ± 13.0 h for the IAI-TXA group, and 63.8 ± 13.6 h for the control group. The times of removal were comparable for the two TXA groups (*P* = 0.13), but were both significantly shorter than that of the control group (*P* < 0.05).

**Table 1:** The demographic data of the subjects in this study

	PAI TXA group (*n* = 49)	IAI TXA group (*n* = 36)	Control group (*n* = 42)	*P* value
Age (years)	68.5 ± 7.4	69.7 ± 7.2	69.6 ± 5.7	0.66
Gender (M/F)	8/41	5/31	10/32	0.48
Height (m)	1.6 ± 0.1	1.6 ± 0.1	1.6 ± 0.1	0.49
Weight (kg)	67.6 ± 11.7	65.1 ± 10.4	67.9 ± 9.3	0.44
BMI (kg/cm^2^)	25.9 ± 3.7	25.6 ± 3.9	26.6 ± 3.8	0.47
Preoperative Hb (g/L)	124.3 ± 10.5	123.8 ± 10.7	125.9 ± 12.9	0.69
INR	0.98 ± 0.2	0.95 ± 0.1	0.94 ± 0.0	0.38
PT (s)	11.5 ± 2.5	11.1 ± 0.7	11.1 ± 0.6	0.33
APTT (s)	27.0 ± 4.3	27.0 ± 3.3	27.0 ± 2.9	0.99
PLT (×10^9^/L)	213.7 ± 77.8	212.1 ± 63.5	212.8 ± 61.1	0.99
Operation time (min)	94.5 ± 16.4	92.1 ± 13.3	88.5 ± 10.3	0.12
Anticoagulant use, % yes	8.2	11.1	9.5	0.90
Thromboembolic tendency, % yes	14.3	11.1	14.3	0.89
Coagulopathies, % yes	2	0	0	0.20

Values are expressed as the mean ± standard deviation

*PAI TXA* peri-articular injection of tranexamic acid, *IAI TXA* intra-articular injection of tranexamic acid, *BMI* body mass index, *Hb* hemoglobin, *INR* international normalized ratio, *PT* prothrombin, *APTT* activated partial thromboplastin time, *PLT* platelet

**Fig. 2:**
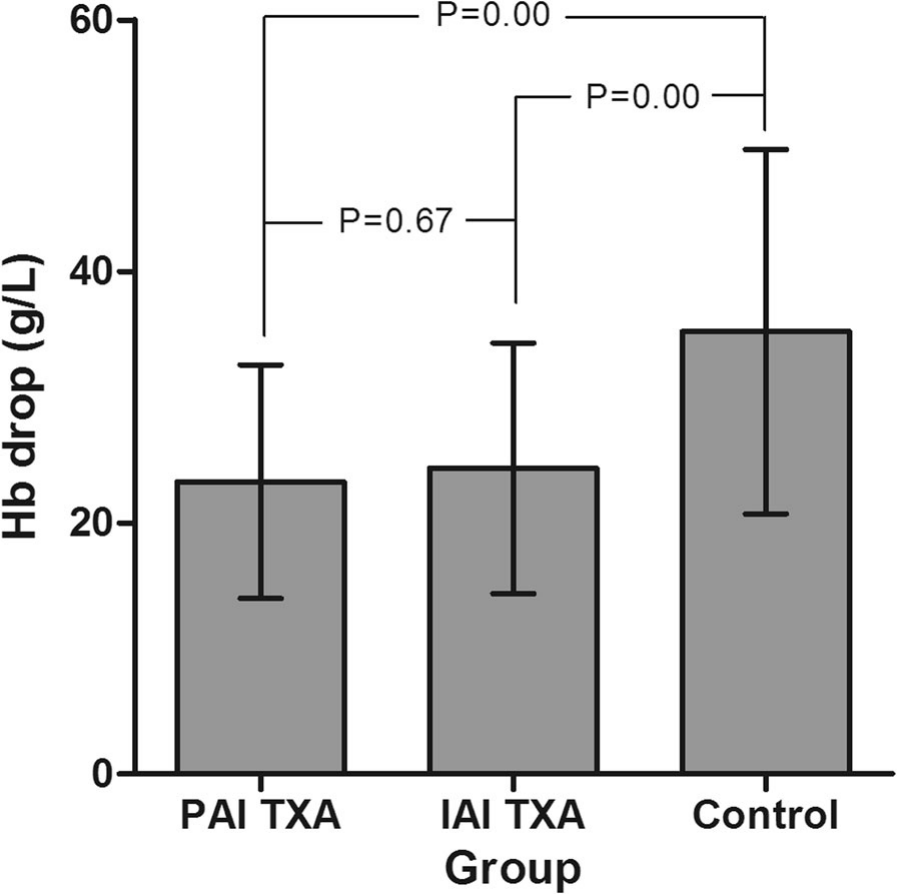
The mean change in Hb concentration before and after surgery of the three groups. Legend: The mean change in Hb concentration was significantly lower for both TXA groups compared to the control group (*P* < 0.05), and had no significant difference between the PAI-TXA and IAI-TXA groups (*P* = 0.67)

The mean change in Hb concentration was significantly lower for both TXA groups compared to the control group (*P* < 0.05), with a mean reduction of the Hb concentration of 23.2 ± 9.3 g/L in the PAI-TXA group, 24.3 ± 10.0 g/L in the IAI-TXA group, and 33.3 ± 14.4 g/L in the control group. The amount of change in Hb concentration was comparable for the PAI-TXA and IAI-TXA groups (*P* = 0.67) (Fig. 2).

**Fig. 3:**
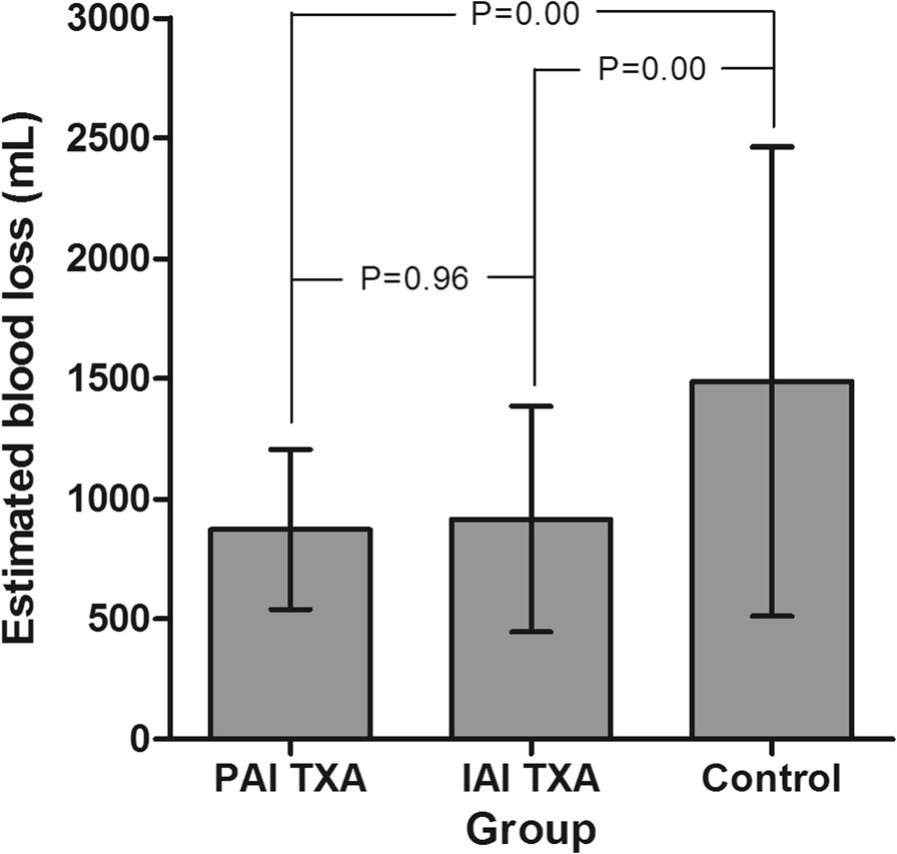
The estimated volume of blood loss of the three groups.Legend: The estimated volume of blood loss was significantly lower for patients in the PAI-TXA and IAI-TXA group compared to those in the control group (*P* < 0.05). There was no significant difference between the two TXA groups (*P* = 0.96)

The estimated volume of blood loss was significantly lower for patients in the PAI-TXA group (872.8 ± 333.3 mL) and IAI-TXA group (914.2 ± 469.4 mL) compared to patients in the control group (1487.1 ± 975.7 mL) (*P* < 0.05). Again, there was no significant difference between the two TXA groups (*P* = 0.96) (Fig. 3).

The number of patients requiring blood transfusions is reported in Table 2. The rate of blood transfusion was comparable for both TXA groups (*P* = 0.71), with 8 patients receiving a transfusion in the PAI-TXA group and 7 patients receiving a transfusion in the IAI-TXA group. In comparison, 16 patients required a blood transfusion in the control group, a transfusion rate that was significantly higher than in the PAI-TXA group, but not significantly different compared to the IAI-TXA group (*P* = 0.07) (Table 2).

**Table 2:** The number of patients requiring blood transfusions in the three groups

Groups	Number of Patients with Transfusions (%)	*P* Value (Compared to PAI TXA Group)	*P* Value (Compared to IAI TXA Group)	*P* Value (Compared to Control Group)
PAI TXA Group (*n* = 49)	8 (16.32)	N/A	0.71	0.02
IAI TXA Group (*n* = 36)	7 (19.44)	0.71	N/A	0.07
Control Group (*n* = 42)	16 (38.10)	0.02	0.07	N/A

*PAI TXA* peri-articular injection of tranexamic acid, *IAI TXA* intra-articular injection of tranexamic acid

We divided the patients who did and did not use anticoagulants into three groups to investigate whether the anticoagulation usage would impact the volume of knee joint drainage, drop in Hb, and estimated blood loss after TKA (Table 3). We found that there was no significant difference between the patients who did and did not use anticoagulation therapy in the PAI, IAI, and control groups (*P* > 0.05). The results were comparable between patients who did not use anticoagulation therapy in the PAI and IAI groups (*P* > 0.05). However, there was a significant difference between these patients and those who did not use anticoagulants in the control group. There was no significant difference among the three subgroups when anticoagulation was used (*P* > 0.05) (Table 3).

**Table 3:** Subgroup analysis of patients on anticoagulants

Anticoagulation use or not	PAI-N (*n* = 45)	PAI-U (*n* = 4)	*P* value	IAI-N (*n* = 32)	IAI-U (*n* = 4)	*P* value	Control-N (*n* = 38)	Control-U (*n* = 4)	*P* value	P*	P**
Drainage volume (mL)	324.7 ± 182.1	327.5 ± 296.0	0.98	299.1 ± 172.7	352.5 ± 148.9	0.56	732.4 ± 289.6	645.0 ± 305.1	0.57	0.00,	0.21,
										0.90,	0.90,
										0.00,	0.18,
										0.00	0.15
Hb drop (g/L)	23.2 ± 8.8	23.5 ± 15.5	0.96	23.9 ± 10.0	28.0 ± 9.8	0.44	32.4 ± 14.0	42.0 ± 18.16	0.21	0.00,	0.24,
										0.80,	0.68,
										0.00,	0.11,
										0.00	0.22
Estimated blood loss (mL)	860.6 ± 343.2	1009.7 ± 150.8	0.40	861.4 ± 457.27	1336.1 ± 337.5	0.06	1486.1 ± 1002.3	1496.5 ± 787.4	0.98	0.00,	0.43,
										0.99	0.40,
										0.00	0.60,
										0.00	0.97

Values are expressed as a mean ± standard deviation

*PAI* peri-articular injection, *IAI* intra-articular injection, *U* anticoagulation used, *N* anticoagulation not used

P*: Differences (Three groups, PAI-N vs. IAI-N, PAI-N vs. Control-N, IAI-N vs. Control-N) among subgroups of PAI-N, IAI-N, and Control-N

P**: Differences (three groups, PAI-U vs. IAI-U, PAI-U vs. Control-U, IAI-U vs. Control-U) among subgroups of PAI-U, IAI-U, and Control-U

There was no DVT or thromboembolic complication in any group. No surgical site or wound complications occurred. All patients had satisfactory ROM results after surgery. At the first three-month follow-up visit, no significant difference was found among the three groups (*P* > 0.05) (Table 4). At this follow-up visit, patients’ ROM reached 120°.

**Table 4:** Follow-up ROM of the patients in the three groups

	One month	Two months	Three months
PAI TXA Group (*n* = 49)	97.8 ± 5.8	110.6 ± 5.2	124.0 ± 4.3
IAI TXA Group (*n* = 36)	96.5 ± 5.1	109.6 ± 4.4	125.4 ± 4.1
Control Group (*n* = 42)	96.9 ± 5.2	108.5 ± 7.4	125.0 ± 4.0
*P* value	0.53	0.22	0.29

Values are expressed as a mean ± standard deviation

*ROM* range of motion, *PAI TXA* peri-articular injection of tranexamic acid, *IAI TXA* intra-articular injection of tranexamic acid

## Discussion

The main findings of the current study were that PAI of TXA is as effective as IAI of TXA in reducing postoperative blood loss during TKA. Both methods had a statistically significant benefit in reducing the change in Hb concentration, volume of joint drainage, and estimated volume of blood loss when compared to a control group.

TXA is a well-known antifibrinolytic agent used to decrease blood loss in various surgical and other medical circumstances, such as in the management of craniocerebral trauma, postpartum hemorrhage, hemophilia hemorrhaging, and menorrhea [20–23]. Recently, IV and topical intra-operative administration of TXA have been reported to reduce postoperative blood loss and the consequent need for blood transfusion after TKA [6, 10, 24]. Several meta-analysis have provided evidence that IV administration of TXA does not increase the risk for DVT [25, 26]; however, Raveendran et al.[27] considered that the effects of TXA on thromboembolic events and mortality remain uncertain. Therefore, topical administration of TXA has been considered a safe alternative to IV administration, limiting the systemic absorption of TXA while providing the benefit of directly increasing drug activity at the application site. Wong et al.[8] found that the plasma levels detected in patients using topical TXA were significantly less than in patients receiving an equivalent IV-administered dose.

Topical TXA has become the routine form of care since Seo et al.[16] reported their findings of the effectiveness of intra-articular administration of TXA in reducing the volume of blood loss and transfusion rate, compared to IV administration. After surgical hemostasis is achieved, a topical application of TXA can reduce topical fibrinolysis and consequently stabilize clot formation and promote microvascular hemostasis, thereby decreasing bleeding [28, 29].

In the majority of studies evaluating the clinical benefits of topical use of TXA, the application method has been an IAI [9–12]. However, the following limitations of IAI of TXA have been noted. In some studies, the volume of intra-articular TXA solution applied ranged only from 5 mL to 25 mL [7, 13, 14]. This low volume of solution may not be sufficient to immerse all soft tissues of the knee joint effectively, particularly when the patient is placed in the supine position for surgery. This would compromise the effectiveness of TXA in controlling bleeding at the sites near the anterior aspect of the knee joint. In addition, in some cases of advanced knee osteoarthritis, extensive soft tissue release is required to achieve soft tissue balance. Such extensive soft release would allow leakage of intra-articular TXA into surrounding tissues. A PAI of TXA, in contrast, would improve permeation of TXA into the deeper soft tissues of the knee joint and avoid TXA leakage. Furthermore, a peri-articular method of administration allows selective application of TXA to potential sites of bleeding, such as released soft tissues and the incisal margin areas in synovial membrane. Finally, a PAI simplifies postoperative management of the patient by eliminating the need to clamp the drain, as required with intra-articular administration.

Postoperative anemia increases the risk for postoperative complications, including wound complications, poor functional recovery, and a longer hospital stay due to the potential need for blood transfusion [3]. In our study, PAI of TXA was effective in lowering the volume of postoperative blood loss and blood transfusion in patients undergoing primary unilateral TKA. PAI-TXA also reduced the volume of postoperative joint drainage, leading to an earlier removal of the drain. The volume of postoperative drainage of 324.9 ± 189.4 mL in patients in the PAI-TXA group, within 48 h, was similar to volumes of 297 ± 196 mL reported by Alshryda et al. (intra-articular) [9] and 401 ± 82.4 mL reported by Roy et al. (intra-articular) [14]. In addition, the effectiveness of PAI-TXA in reducing the volume of postoperative blood loss and preserving Hb concentrations in our study is consistent and comparable with the findings of previous studies reported by Georgiadis et al. (intra-articular) [6], Wong et al. (intra-articular) [8], and Aguilera et al. (intravenous) [30].

The method for topical administration of TXA, however, has remained a controversial issue in clinical practice. Maniar et al.[15] evaluated five different methods of TXA administration, reporting that an IAI, compared to no intervention, reduced the volume of blood loss but had no discernible effects on postoperative joint drainage volume. Sarzaeem et al.[31] compared IV administration of TXA to two topical methods, IAI and peri-articular irrigation (not injection), to a control group. Among the TXA groups, IAI of TXA led to less blood loss compared to IV administration, and IV administration led to less blood loss than irrigation of the knee joint with TXA. In our study, we found that both PAI and IAI of TXA significantly reduced the blood loss and wound drainage after TKA compared to the control group.

TXA is a synthetic analog of the amino acid lysine, and it serves as an antifibrinolytic. Although few adverse effects have been reported in the orthopedic field, concerns about thromboembolism persist. Many studies show that IV or IAI of TXA during TKA was not associated with a significant incidence of DVT [7, 8, 14, 16]. In the current study, no DVT or thromboembolic complication was found, confirming the results of those studies.

Anticoagulant drugs may influence bleeding. In our study, we divided each group into two subgroups, the anticoagulation not used subgroup and the anticoagulation used subgroup, in order to investigate the impact of anticoagulation use on the volume of knee joint drainage, drop in Hb, and estimated blood loss. There was no significant difference among the three anticoagulation used subgroups (*P* > 0.05), which were inconsistent with the results among the three groups; this may be attributed to the small sample size of these three subgroups, which only had 4 patients each. Further well-designed studies may be needed to understand if anticoagulant drug use would influence the therapeutic effect of TXA.

The limitations of our study need to be acknowledged. Foremost, as a retrospective study with a relatively small sample size, the level of evidence is low. In addition, we did not measure systemic blood levels of TXA and, therefore, the level of TXA leaked from the soft tissue and joint space of the knee into the systemic circulation could not be determined. Furthermore, avoiding TXA leakage is thought to be a theoretical advantage of the PAI method. In our experience, no TXA leakage occurred using this method. Due to our small sample size, further well-designed randomized controlled trials will be required to investigate if avoiding TXA leakage is an actual advantage of the PAI method compared to the IAI method,

## Conclusions

The peri-articular injection of TXA is as effective as the intra-articular injection in reducing postoperative blood loss in TKA, while possessing several potential advantages.

## Abbreviations

DVT: Deep venous thrombosis
Hb: Hemoglobin
IAI-TXA: Intra-articular injection of TXA
PAI-TXA: Peri-articular injection of TXA
ROM: Range of motion
TKA: Total knee arthroplasty
TXA: Tranexamic acid
